# Neuropeptide Initiated Mast Cell Activation by Transcutaneous Electrical Acupoint Stimulation of Acupoint LI4 in Rats

**DOI:** 10.1038/s41598-018-32048-3

**Published:** 2018-09-17

**Authors:** Li-Zhen Chen, Yu Kan, Zhi-Yun Zhang, Yi-Li Wang, Xiao-Ning Zhang, Xiao-Yu Wang, Wei He, Xiang-Hong Jing

**Affiliations:** 1 Research Center of Meridians, Institute of Acupuncture and Moxibustion, China Academy of Chinese Medical Sciences, Beijing, 100700 China; 2 Department of Neurobiology and Acupuncture Research, The Third Clinical Medical College, Zhejiang Chinese Medical University, Hangzhou, 310053 China

## Abstract

Transcutaneous electrical acupoint stimulation (TEAS) has been consistently used clinically for its ease of operation, non-invasiveness and painlessness, in contrast to the characteristics of inserted needles. However, the mechanism remains unknown. The aim of this study was to investigate the local response of TEAS at Hegu acupoint (LI4). Immunohistochemistry was used to measure the expression of tryptase-positive mast cells, neuropeptides of the calcitonin gene-related peptide (CGRP) and substance P (SP) in LI4. Mast cells were also labelled with serotonin (5-HT), neurokinin-1 receptor (NK-1R) and toluidine blue. The results showed that cutaneous CGRP and SP immune-positive (CGRP-IP or SP-IP) nerve fibres in LI4 were more highly expressed. There were high degrees of mast cell aggregation and degranulation with release of 5-HT near the CGRP-IP or SP-IP nerve fibres and blood vessels after TEAS. The degranulation of mast cells (MCs) was accompanied by expression of NK-1R after TEAS. Either mast cell membrane stabilizer (Disodium cromoglycate) or NK-1R antagonist (RP 67580) diminished the accumulation and degranulation of MCs induced by TEAS. Taken together, the findings demonstrated that TEAS induced sensory nerve fibres to express CGRP and SP, which then bound to the NK-1R on MCs, after which MCs degranulated and released 5-HT, resulting in TEAS-initiated acupuncture-like signals.

## Introduction

Acupuncture is a medical technique that involves insertion of needles into acupoints to relieve pain and regulate functions of internal organs. Because of its curative effects and few side effects, acupuncture has been practiced in more than 183 countries and areas. Studies showed that the therapeutic effects of acupuncture were mediated by nervous system^1–3^, immune system^4^ and other systems.

There are several manipulation methods used to stimulate acupoints, including acupressure, manual acupuncture (MA) and electroacupuncture (EA). Although MA is widely used for its ease of manipulation and rapid efficiency, it depends highly on the skill of the acupuncturist and is difficult to duplicate. EA became popular for its adjustable strength, frequency and easy quantification in the clinic. However, some people are afraid of the painful sensation of puncturing, and the puncture method is highly dependent on the doctor’s technique.

Transcutaneous electrical acupoint stimulation (TEAS) combines the transcutaneous electrical stimulation of physical therapy with acupoint therapy but less invasive than MA^5^. Increasing evidence supports the notion that TEAS is effective in reducing pain during labour^6^, treating reproductive disorders^7^, preventing and treating nausea and vomiting in patients receiving electroconvulsive therapy^8^, and improving the pregnancy rate and implantation rate in patients with implantation failure^9^. Transcutaneous electrical nerve stimulation (TENS) or TEAS instead of MA or EA is becoming a trend for they had same effect on antinociception and shares common neural mechanisms^10^. Our previous study demonstrated that local cutaneous nerve terminals and mast cells (MCs) responded to MA. MA at acupoint LI4^11^ induces higher expression of neuropeptides of calcitonin gene-related peptide (CGRP) and substance P (SP) in subepidermal nerve fibres to activate MCs. Whether nerve-mast cell cross-talk contributes to the effects of TEAS remains unclear. The present study focused on local histologic and cellular changes at LI4 after TEAS. We hypothesized that TEAS activated cutaneous sensory nerve fibres to express sensory CGRP and SP, and that SP bonded to neurokinin-1 receptor (NK-1R) situated in MCs to initiate MC release of tryptase and 5-HT.

## Material and Methods

### Ethical approval

All experimental protocols reported here were in accordance with the National Institutes of Health Guide for the Care and Use of Laboratory Animals (NIH Publications No. 80-23) revised in 1996. It also conformed to the Animal Use and Care of Medical Laboratory Animals from the Ministry of Public Health of the People’s Republic of China. All experiments were carefully conducted according to the ethical guidelines for the use of experimental pain in conscious animals published by the International Association for the Study of Pain^12^. The study also obtained ethics committee approval from the Institutional Animal Welfare and Use Committee of IAM-CACMS (No. 20170313).

### Animals and TEAS Application

The animals were purchased from the Institute of Laboratory Animal Sciences, China Academy of Medical Sciences (experimental animal license number: SCXK(Jing)2014-0013). Rats were housed in standard animal facilities in which the room temperature was maintained at 24 ± 2 °C, the humidity was 60–70%, and the noise levels were lower than 60 dB. All animals were grouped in twos or threes with ad libitum access to food and water. The bedding material and drinking water were replaced every day to keep the cages clean and dry. The animals were maintained on a standard 12-hour light-dark cycle (dark cycle 8:00 PM-8:00 AM) and were allowed to acclimate to the housing conditions for seven days prior to the experiment.

**Figure 1:**
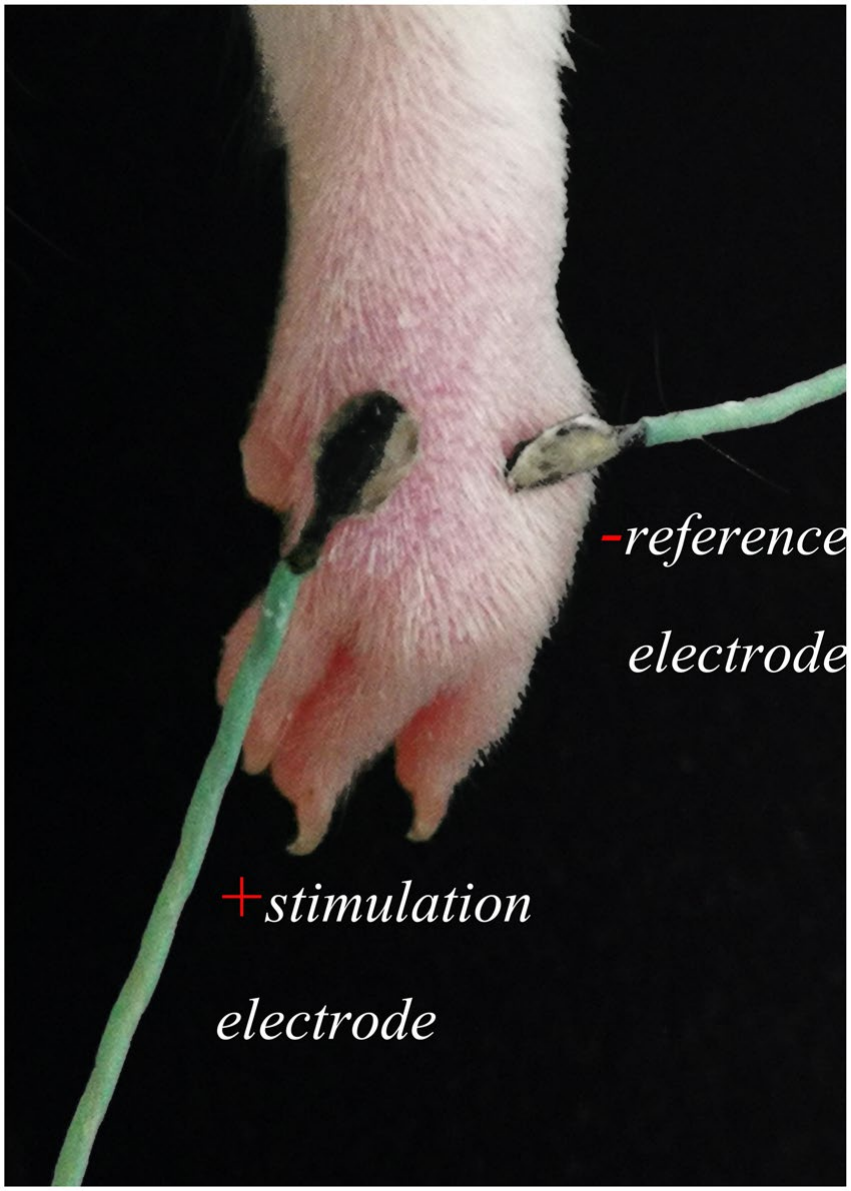
TEAS applied at the LI4 acupoint in a rat.

The experiment was performed on 15 adult male Sprague-Dawley rats with weights of 180–220 g, Under anaesthesia with 10% urethane (1 g/kg), TEAS was applied at the right Hegu acupoint (LI4), which is located on the radial side at the midpoint of the second metacarpal bone, as shown in Fig. 1. The parameters applied were a frequency of 2/100 Hz and a current of 10 mA to cause obvious shaking of the front paws for 20 min, consistent with those parameters used in routine clinical treatment or basic research^6,11^. The contralateral LI4 of the same rat was used as the non-stimulated control.

### Administration of mast cell membrane stabilizer or NK-1R antagonist

In order to confirm if NK-1R or mast cell was activated by TEAS, mast cell membrane stabilizer Disodium cromoglycate (DC) (C0399-1G, Sigma-Alorich, 50 mg/kg) or NK-1R antagonist (RP 67580, sc-204894, Santa Cruz Biotechnology, 10 mg/kg) was administrated by intraperitoneal injection 30 min before TEAS^13,14^.

### Tissue preparation and immunohistochemical staining

After TEAS, the anaesthetized rats were immediately perfused with 200 mL of 0.9% saline, followed by 250 mL of cold 4% paraformaldehyde in 0.1 M phosphate-buffered saline (PBS, pH 7.4). The skin tissue in the LI4 area (approximately 2 × 2 × 1 mm^3^, approximately 9 mg) was dissected. The collected tissues were post-fixed in 4% paraformaldehyde at 4 °C for 4 hours and cryoprotected in phosphate-buffered 25% sucrose at 4 °C for 24 hours. Following post-fixation, the skin was embedded in artificial medium (Shandon Cryomatrix™, 120 mL, Thermo Scientific, USA), frozen, and cut into 20-μm sections on a cryostat (Thermo, Microm International FSE, Germany). The sections were then thaw-mounted on SuperFrost® Plus slides (Thermo Scientific, USA) and allowed to dry. The sequentially mounted slides were prepared for various kinds of fluorescence immunohistochemical and histochemical staining. The primary antibodies were as follows: mouse monoclonal anti-MC tryptase antibody (1:1500, Abcam, England), rabbit polyclonal anti-SP antibody (1:500, Abcam), mouse monoclonal anti-CGRP antibody (1:500, Abcam), goat polyclonal anti-5-HT antibody (1:2000, Abcam), and goat polyclonal anti-NK-1R antibody (1:50, Santa Cruz, USA). Information about all the primary antibodies and the isotype controls are listed in Table 1.

**Table 1:** Information on the primary antibodies used in this study.

Antibody	Host Species	Antibody Code	Isotype control	Company
Anti-MC Tryptase	Mouse	ab2378	ab170190	Abcam, England
Anti-CGRP	Mouse	ab81887	ab18415	Abcam, England
Anti-SP	rabbit	ab67006	ab172730	Abcam, England
Anti-5-HT	goat	ab66047	ab37373	Abcam, England
Anti-NK1R	goat	Sc14116	Sc2028	Santa Cruz, USA

After an initial wash in 0.1 M PBS (pH 7.4), the tissues were preincubated in a solution of 3% normal goat or donkey serum and 0.5% Triton X-100 in 0.1 M phosphate-buffered solution (PB, pH 7.4) for 30 min to block non-specific binding. The sections were then incubated with primary antibodies for 24 hours at 4 °C. After washing in 0.1 M PB for 3–10 min, goat anti-mouse Alexa Fluor 488 or 594 secondary antibody (1:1000, Molecular Probes, Eugene, Oregon, USA), goat anti-rabbit Alexa Fluor 488 or 594 secondary antibody (1:1000; Molecular Probes), donkey anti-rabbit Alexa Fluor 488 or 594 secondary antibody (1:1000; Molecular Probes), donkey anti-goat Alexa Fluor 488 or 594 secondary antibody (1:1000; Molecular Probes), and donkey anti-mouse Alexa Fluor 488 or 594 secondary antibody (1:1000; Molecular Probes) were used to visualize the corresponding primary antibodies. Additionally, Alexa Fluor 488 phalloidin (1:1000, Molecular Probes) was used to counterstain the cytoskeleton, and 4′,6-diamidino-2-phenylindole, dihydrochloride (DAPI, 1:40000; Molecular Probes) was used to counterstain the nuclei. Following a final wash in 0.1 M PBS, slides were coverslipped with PBS–glycerol. Negative controls were also performed by leaving out the primary antibodies during the staining procedure. The double immunohistochemical staining was to examine the relationship between 1) SP immune-positive (SP-IP) nerve fibres and CGRP (CGRP-IP) nerve fibres, 2) MCs and CGRP-IP nerve fibres, 3) MCs and SP-IP nerve fibres, 4) MCs and blood vessels (phalloidin), 5) 5-HT expression on MCs, 6) 5-HT and blood vessels (phalloidin), 7) 5-HT and SP-IP nerve fibres, and 8) MCs and NK-1R. The immunohistochemical staining for each rat was performed at the same time to ensure staining consistency.

### Toluidine blue staining

The sections were also stained by toluidine blue to label MCs. The sections were first stained with 0.1% toluidine blue (formulated with distilled water) for approximately 15 seconds and then quickly washed with distilled water three times to remove floating colour. Next, 30% alcohol (formulated with PB) was used to separate colours, and sections were dehydrated by sequentially dipping them quickly (approximately 5 seconds) in 75%, 85%, 95% and 100% alcohol. Finally, sections were dipped in xylene for 5 minutes and mounted with neutral resin sheet. After staining, randomly selected sections were examined under light microscopy. An intact MC showed deep blue staining; while degranulated MC showed purple or red staining with granules extruded adjacent to the MC. MC degranulation was determined as described previously^15^.

### Observation and Data Analysis

Slides were observed on a confocal imaging system (FV1200, Olympus, Japan). Olympus Image Processing Software was used by an investigator who was blinded to the staining to analyse the images. The three channels (Alexa 594, Alexa 488 and DAPI-stimulated light) were selected to correspond with the staining specimens, and the intensity of the 3 light intensities was adjusted and balanced while taking photographs. Images were collected in successive frames (Z series) of each 1.5 μm section and were integrated into a single in-focus image. The summed lengths of CGRP or SP immune-positive nerve fibres from 5 randomized sections of each rat were calculated within a magnified field (×400) and averaged for analysis. The numbers of tryptase-positive MCs and 5-HT-positive MCs were counted within a magnified field (×400) in 5 randomized sections from each group of rats. Data were expressed as the mean ± SD and were processed with the statistical software package GraphPad Prism 5.0.

## Results

### TEAS induced both CGRP and SP immune-positive nerve fibres highly expressed in the epidermis and dermis of acupoint LI4

**Figure 2:**
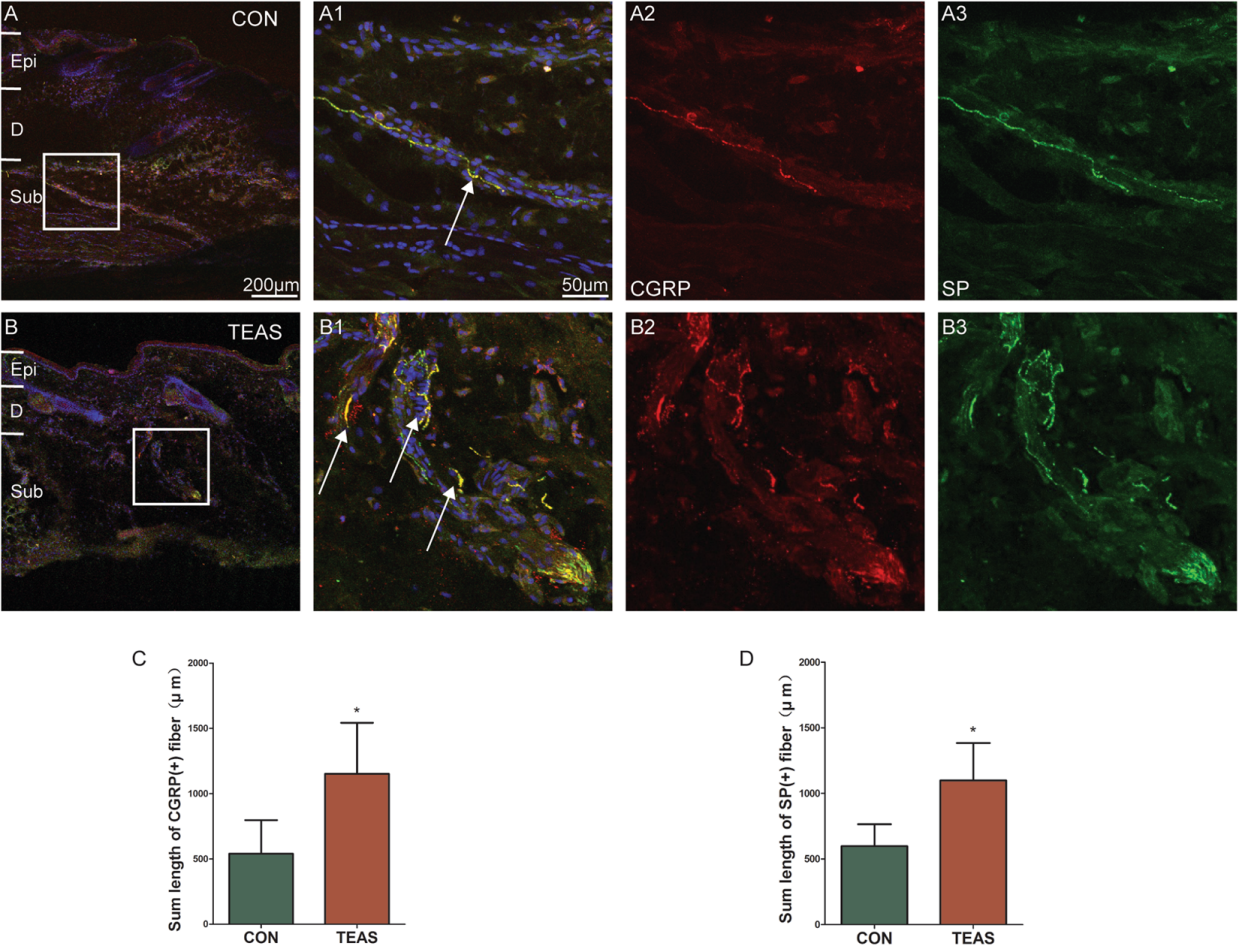
Expression of SP-IP and CGRP-IP nerve fibres in the area of LI4 labelled by fluorescent immunohistochemistry for CGRP (red), SP (green), and DAPI (blue). (**A**,**B**) SP-IP and CGRP-IP nerve fibres located in intra-epidermis and dermis. (**A**) SP-IP and CGRP-IP nerve fibres in the control. (**B**) Numerous SP-IP and CGRP-IP nerve fibres were present after TEAS. A1 and B1 were the magnified images from the boxed areas of (**A**) and (**B**), respectively. A2–A3, B2–B3 represented CGRP (A2, B2) and SP (A3, B3) in figures A1 and B1, respectively. Double-labelling of SP-IP and CGRP-IP nerve fibre was in yellow (white arrow). (**C** and **D**): The summed lengths of CGRP-IP and SP-IP nerve fibres were greater after TEAS (*P < 0.05 *vs* CON). A scale bar for figures (**A**) and (**B**) was shown in (**A**), and for figures A1–3 and B1–3 was in A1. Epi: epidermis, D: dermis, Sub: subcutaneous tissue.

CGRP and SP immune-positive nerve fibres (CGRP-IP or SP-IP) were distributed in the intra-epidermis and dermis at acupoint LI4 (Fig. 2A1). Co-expression of CGRP-IP nerve fibres and SP-IP nerve fibres was also observed (Fig. 2A1–A3). Compared to the control, the summed lengths of CGRP-IP nerve fibres were greater after TEAS (CON: 540.1 ± 257.2 μm *vs* TEAS: 1151 ± 391.4 μm, *P* = 0.0267) (Fig. 2A2,B2,C), and the summed lengths of SP-IP nerve fibres were greater after TEAS (CON: 597.8 ± 166.8 μm *vs* TEAS: 1099 ± 284.7 μm, *P* = 0.0146) (Fig. 2A3,B3,D).

### TEAS induced MCs aggregation near nerve fibres and blood vessels with degranulation of 5-HT at acupoint LI4

**Figure 3:**
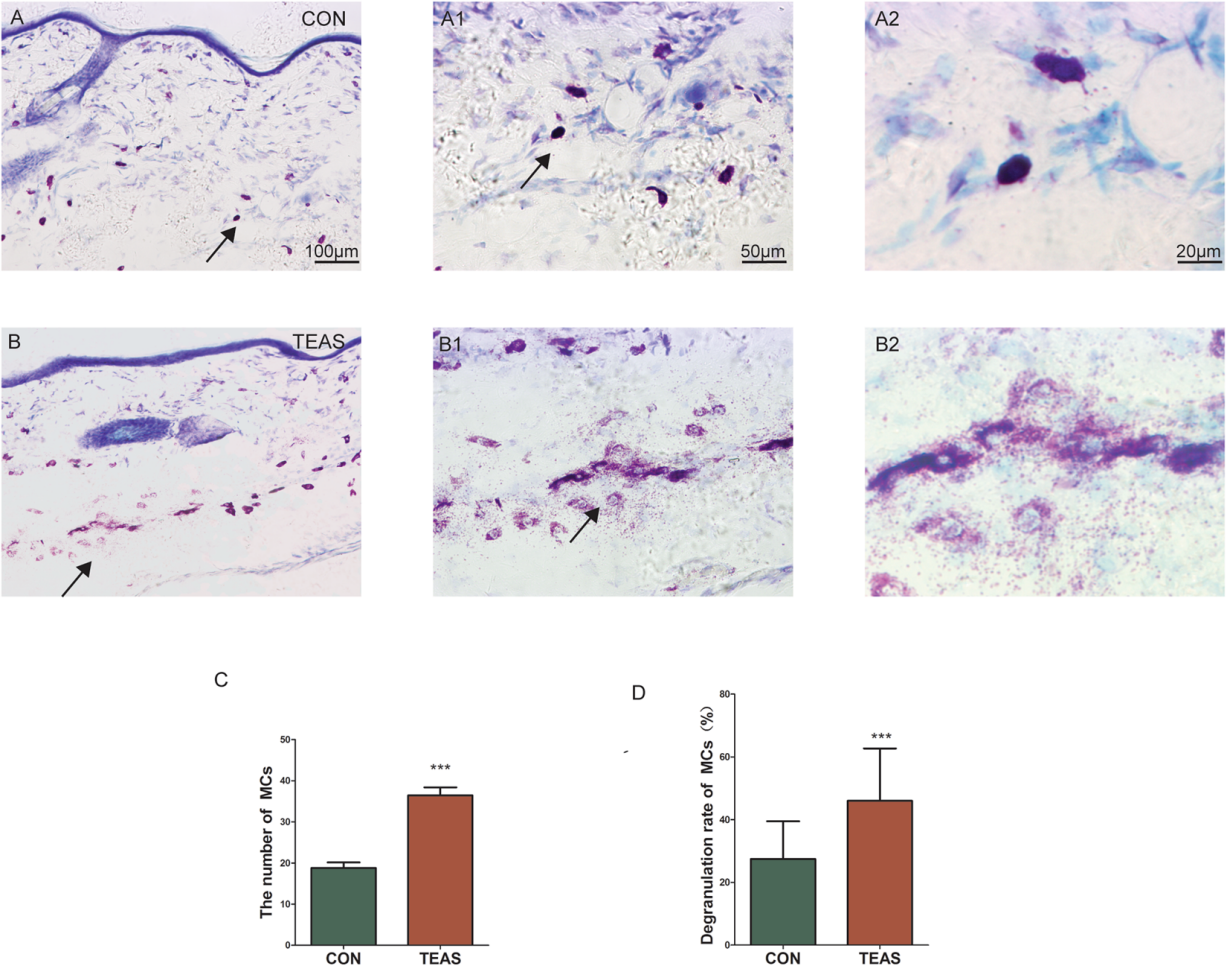
Toluidine blue-stained MCs scattering in LI4. A, A1, A2, B, B1, B2: After TEAS, MCs aggregated markedly with increased volume and some ruptured to release granules. A1 and B1 are the magnified images from the areas (marked with black arrow) of (**A**) and (**B**), respectively. A2 and B2 are the magnified images from the areas (marked with black arrow) of A1 and B1, respectively. (**C**,**D**) The number and degranulation rate of MCs significantly increased after TEAS (***P < 0.001 *vs* CON).

**Figure 4:**
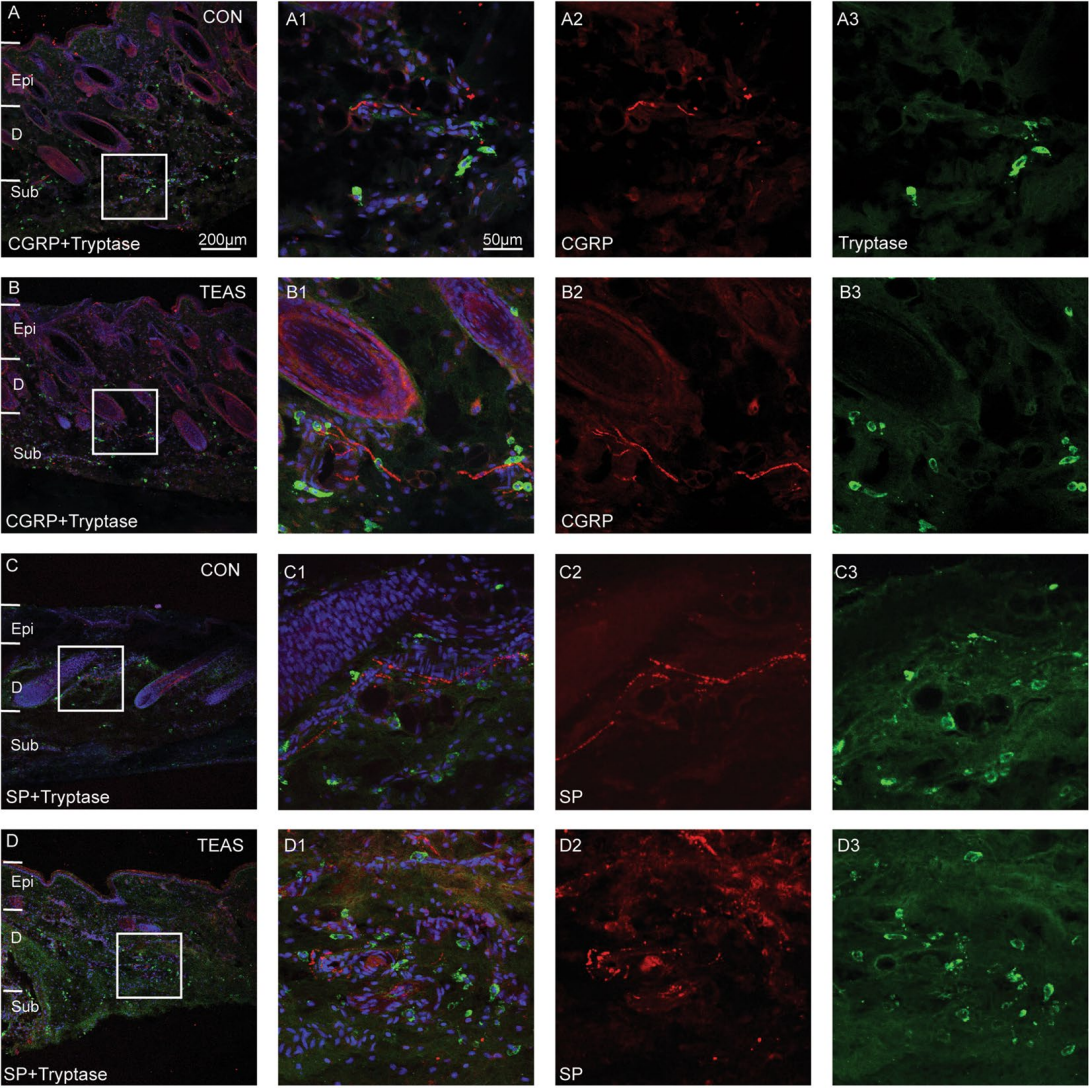
Triple-staining was conducted to label MCs with tryptase (green), SP-IP or CGRP-IP nerve fibres (red), and nuclei with DAPI (blue). (**A**–**D**) MCs were around the SP-IP or CGRP-IP nerve fibres in dermis and subcutaneous tissue in CON (**A**,**C**) and TEAS (**B**,**D**) of LI4. Numerous SP-IP or CGRP-IP nerve fibres were present after TEAS (B2, D2). A1, B1, C1 and D1 were magnified images from the boxed areas of (**A**–**D**). A2–3, B2–3, C2–3 and D2–3 represented CGRP (A2, B2), SP (C2, D2) or tryptase (A3, B3, C3, D3). MCs aggregated and degranulated with broken granules after TEAS (D3). Epi: epidermis, D: dermis, Sub: subcutaneous tissue.

For historical analysis of dermal MCs, sections at acupoint LI4 were stained with toluidine blue. Normally, toluidine-labelled cells appeared as intact cells and were dyed deep blue (Fig. 3A). After TEAS, MCs aggregated markedly and the granules presented purple-red metachromasia with swollen shapes. The number of MCs increased after TEAS (CON: 18.80 ± 5.983 *vs* TEAS: 36.45 ± 8.610, *P* = 0.00008). Some even ruptured to release granules (Fig. 3B). The degranulation rate also increased after TEAS (CON: 27.47% ± 12.02% *vs* TEAS: 46.03% ± 16.68%, *P* = 0.0002). Tryptase-positive MCs were also stained by using the immunofluorescence technique. Normally, tryptase-positive MCs were distributed in the dermis and maintained an intact shape at LI4 (Fig. 4A,C). After TEAS, MCs aggregated around the CGRP-IP or SP-IP nerve fibres at LI4 and degranulated (Fig. 4B,D).

**Figure 5:**
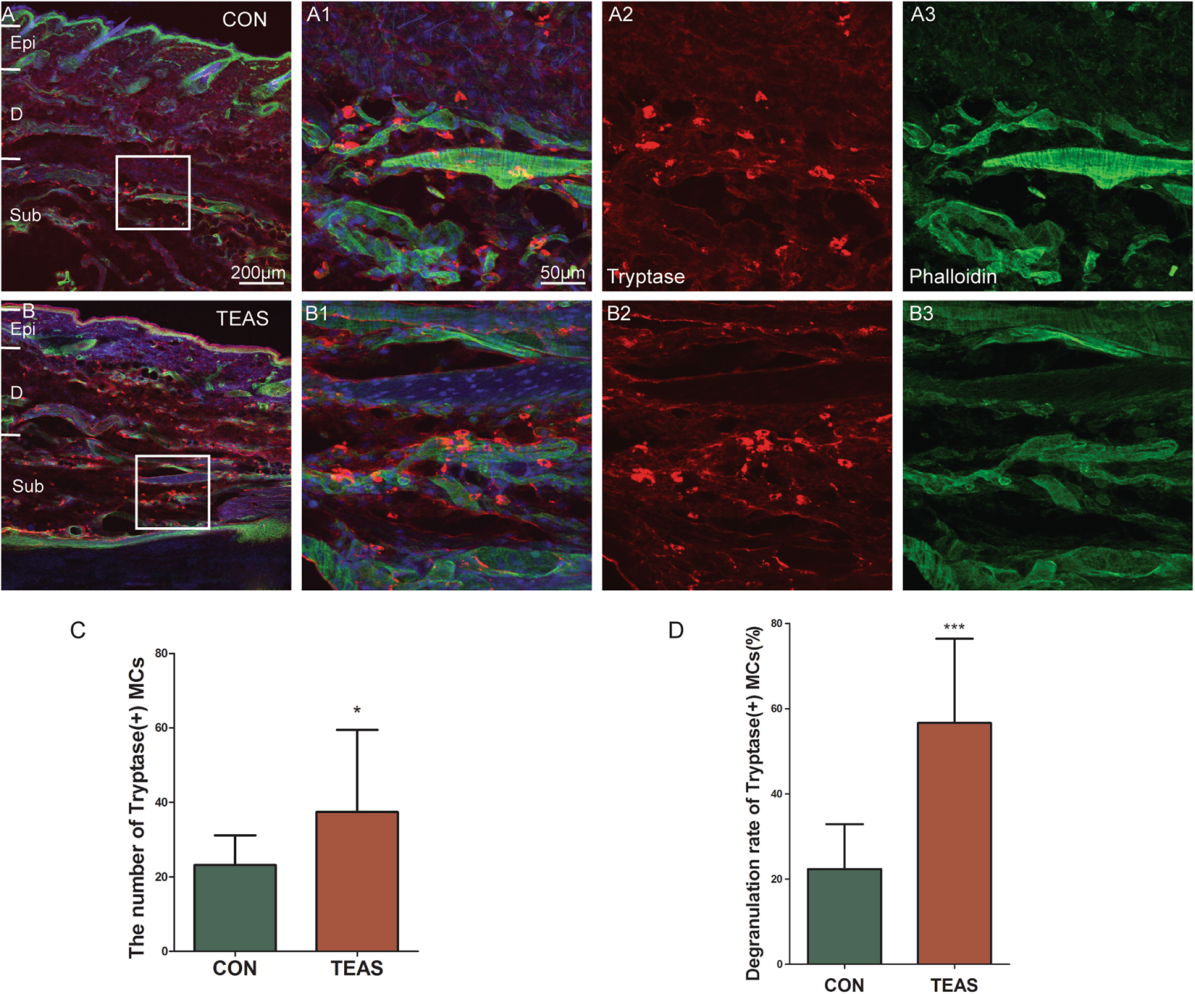
TEAS-induced MC aggregation and degranulation. Triple-staining was conducted to label MCs with tryptase (red), blood vessels with phalloidin (green), and nuclei with DAPI (blue). (**A**,**B**) MCs located around blood vessels in the dermis and subcutaneous tissue in CON (**A**) and TEAS (**B**). A1 and B1 were magnified images from the boxed areas of A and B, which was an overlapped picture to show MCs and blood vessels. A2–3 and B2–3 were single-labelled by tryptase (A2, B2) and phalloidin (A3, B3), respectively. Granules were released from MCs after TEAS (B2). (**A**) scale bar for figures A and B was marked in A, and for figures A1–A3 and B1–B3 is in A1. (**C**,**D**) The number and degranulation rate of MCs markedly increased after TEAS. (*P < 0.05, ***P < 0.001 *vs* CON) Epi: epidermis D: dermis. Sub subcutaneous tissue.

**Figure 6:**
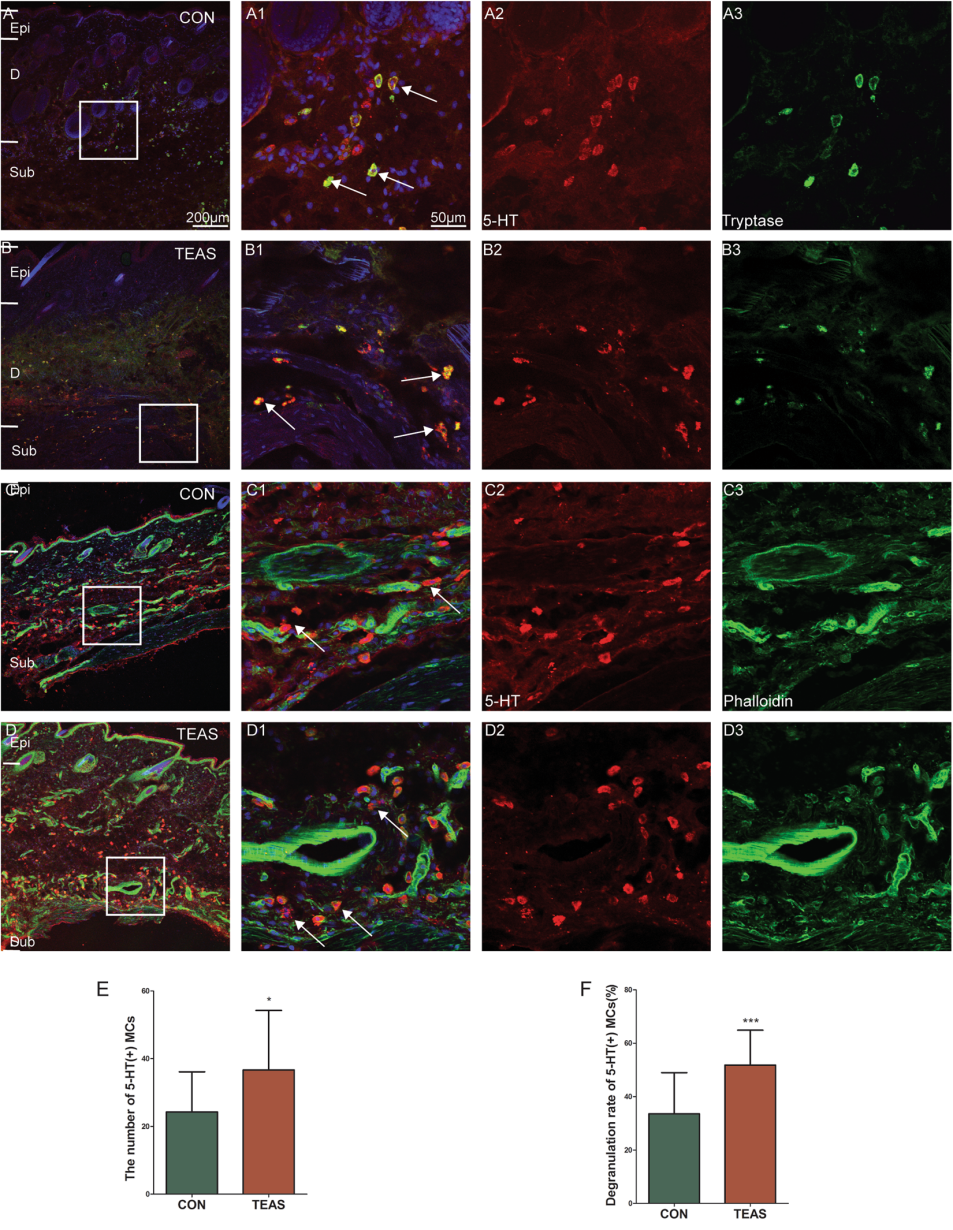
TEAS-induced 5-HT release from degranulated MCs near blood vessels. Triple-staining was conducted to label cells with 5-HT (red), tryptase/phalloidin (green), and nuclei with DAPI (blue). (**A**), A1–A3: both tryptase and 5-HT were co-expressed in cells in the dermis and subcutaneously at LI4 (A1). Double-labelled cells are indicated by the white arrow. There are also some single-labelled cells with similar size. (**B**) B1–B3: TEAS induced MCs degranulation and the granules co-expressed 5-HT and tryptase. And, 5-HT-labelled cells located around blood vessels in the dermis and subcutaneous tissue. (**C**) C1–C3: CON; (**D**) D1–D3: TEAS. A scale bar for figures (**A**–**D**) was marked in (**A**), and (**A**) scale bar for other figures was marked in A1. (**E**,**F**) The number and degranulation rate of 5-HT cells markedly increased after TEAS. (*P < 0.05, ***P < 0.001 *vs* CON) Epi: epidermis. D: dermis. Sub: subcutaneous tissue.

Tryptase-positive MCs were primarily distributed in the dermis in the control LI4 (Fig. 5A). After TEAS, MCs aggregated near blood vessels (Fig. 5B1). Compared with the non-stimulated control at LI4, the number of MCs (CON: 23.15 ± 8.015 *vs* TEAS: 37.40 ± 22.06, *P* = 0.0371) and degranulation rates (CON: 22.35% ± 10.59% *vs* TEAS: 60.55% ± 15.46%, *P* = 0.00002) were markedly higher (Fig. 5C,D, *P < 0.05, ***P < 0.001). Degranulation of MCs was followed by the release of 5-HT^16^. To confirm whether 5-HT was expressed in MCs, both tryptase and 5-HT were double-stained in the same slice. Normally, both tryptase and 5-HT were co-expressed in some cells in the dermis and subcutaneously (Fig. 6A). Some 5-HT immune-positive cells were also located near blood vessels (Fig. 6C). Furthermore, the sizes of tryptase- and 5-HT-positive cells were similar. After TEAS, MCs degranulated, and the granules co-expressed 5-HT and tryptase (Fig. 6B). TEAS also promoted 5-HT immune-positive cell aggregation near blood vessels (Fig. 6D). The number of 5-HT immune-positive cells significantly increased after TEAS (CON: 24.30 ± 11.84 vs TEAS: 36.70 ± 17.56, *P* = 0.0184) (Fig. 6E *P < 0.05). The ratio of degranulation of 5-HT-positive cells after TEAS was also significantly higher than that of the control (CON: 33.50 ± 15.37 *vs* TEAS: 51.90 ± 13.16, *P* = 0.0008) (Fig. 6F ***P < 0.001).

### TEAS induced SP -initiated MCs activation via NK-1R at acupoint LI4

**Figure 7:**
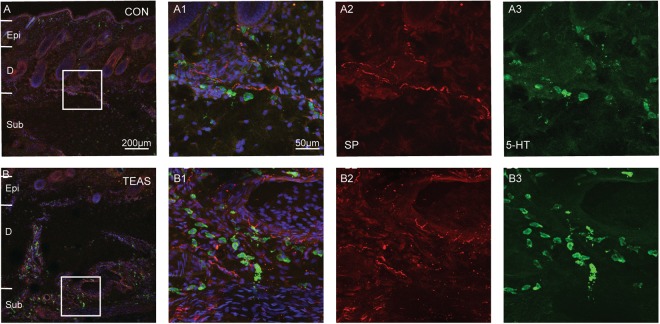
Triple-staining was conducted to label cells with 5-HT (green), nerve fibres with SP (red), and nuclei with DAPI (blue). TEAS-induced SP-IR fibres and 5-HT immune-positive cells increased at LI4 (B1–3). Epi: epidermis, D: dermis, Sub: subcutaneous tissue.

**Figure 8:**
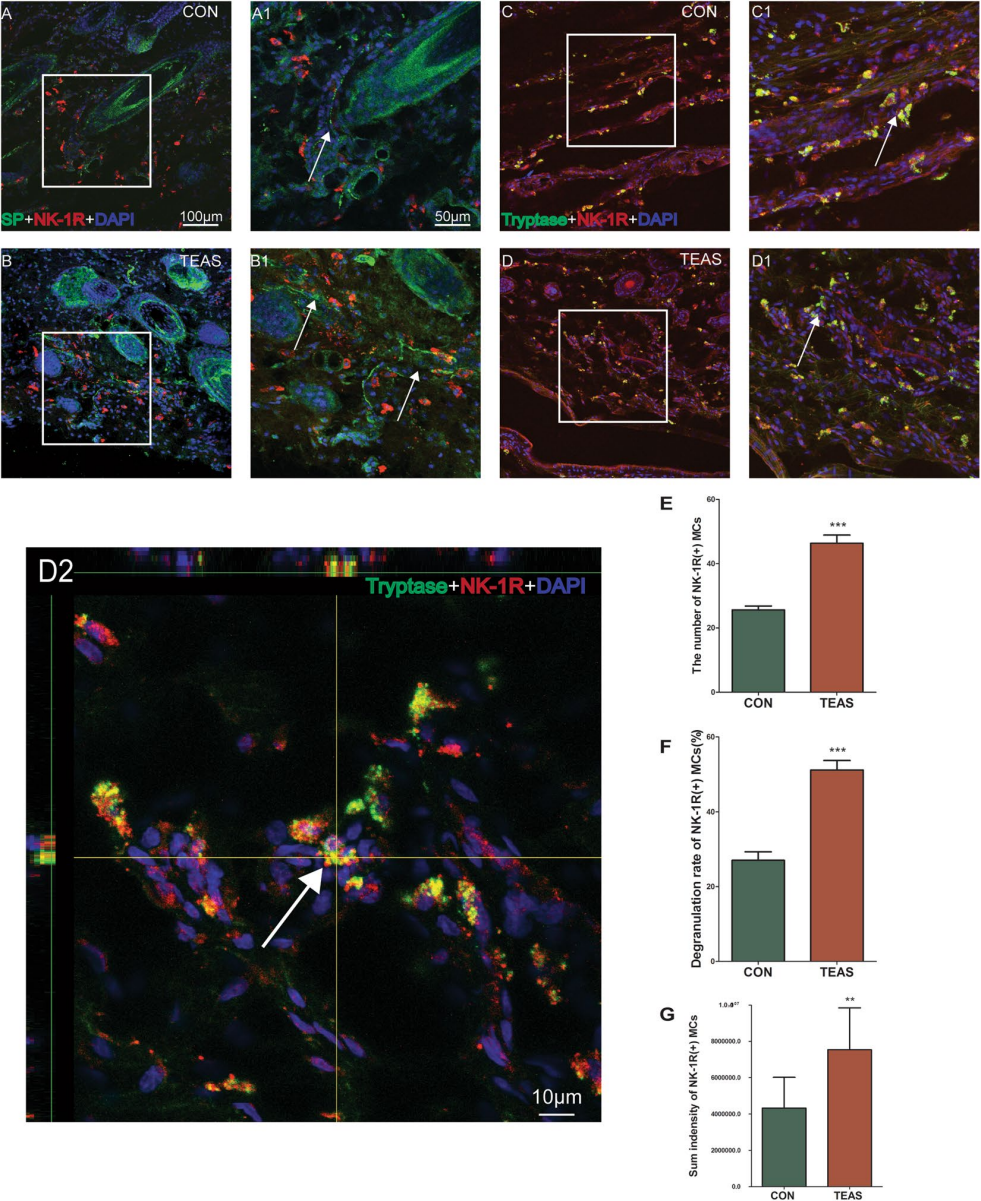
Triple-staining was conducted to label SP-IP nerve fibres/tryptase (green), NK1R (red) with nuclei (DAPI, blue) to confirm whether MCs were activated by SP through NK-1R. (**A**,**B**) Triple-staining of SP-IP nerve fibres, NK-1R and DAPI. A1 and B1 were the magnified images from the areas (white boxes) of (**A**) and (**B**) respectively. (**C**,**D**) Triple-staining of tryptase, NK-1R with DAPI. C1 and D1 were the magnified images from the areas (white boxes) of (**C**) and (**D**), respectively. Double-labelled cells were in yellow with clear outline in control (C1, white arrow). TEAS induced MCs activation with marked aggregation (D1, white arrow), swollen volume, deformation, and rupture to release granules (D2). A scale bar for (**A**–**D**) is shown in A, and that for A1, B1, C1, and D1 is shown in A1. (**E**,**F** and **G**) The number, degranulation rate and summed intensity of NK-1R on MCs markedly increased after TEAS. (**P < 0.05 *vs* CON, ***P < 0.001 *vs* CON).

We further explored the relationship between SP and MCs induced by TEAS. Normally, SP-IP nerve fibers were distributed in the epidermis, dermis and subcutaneous tissue. 5-HT labeled cells were distributed in the dermis and subcutaneous tissue (Fig. 7A–A3). After TEAS, SP-IP nerve fibers expressed more highly than that of the control (Fig. 2). Moreover, MCs gathered around SP-IP nerve fibers with degranulation of 5-HT (Fig. 7B–B3). Meanwhile NK-1R was one of the SP receptors expressed in MCs^17^. After TEAS, NK-1R was highly expressed around SP-IP nerve fibres and was also highly expressed in MC granules. The numbers (CON: 25.65 ± 5.24 *vs* TEAS: 46.35 ± 11.38, *P* = 0.00004), degranulation rates (CON: 27.03% ± 10.18%; TEAS: 51.17% ± 11.25%, *P* = 0.00003) and summed intensities (CON: 4.326 × 10^6^ ± 1.695 × 10^6^; TEAS: 7.543 × 10^6^ ± 2.302 × 10^6^, *P* = 0.0026) of NK-1R on MCs markedly increased after TEAS (Fig. 8), suggesting that the NK-1R and SP cross-talked after TEAS.

### The activation of MCs induced by TEAS was diminished by specific antagonists

**Figure 9:**
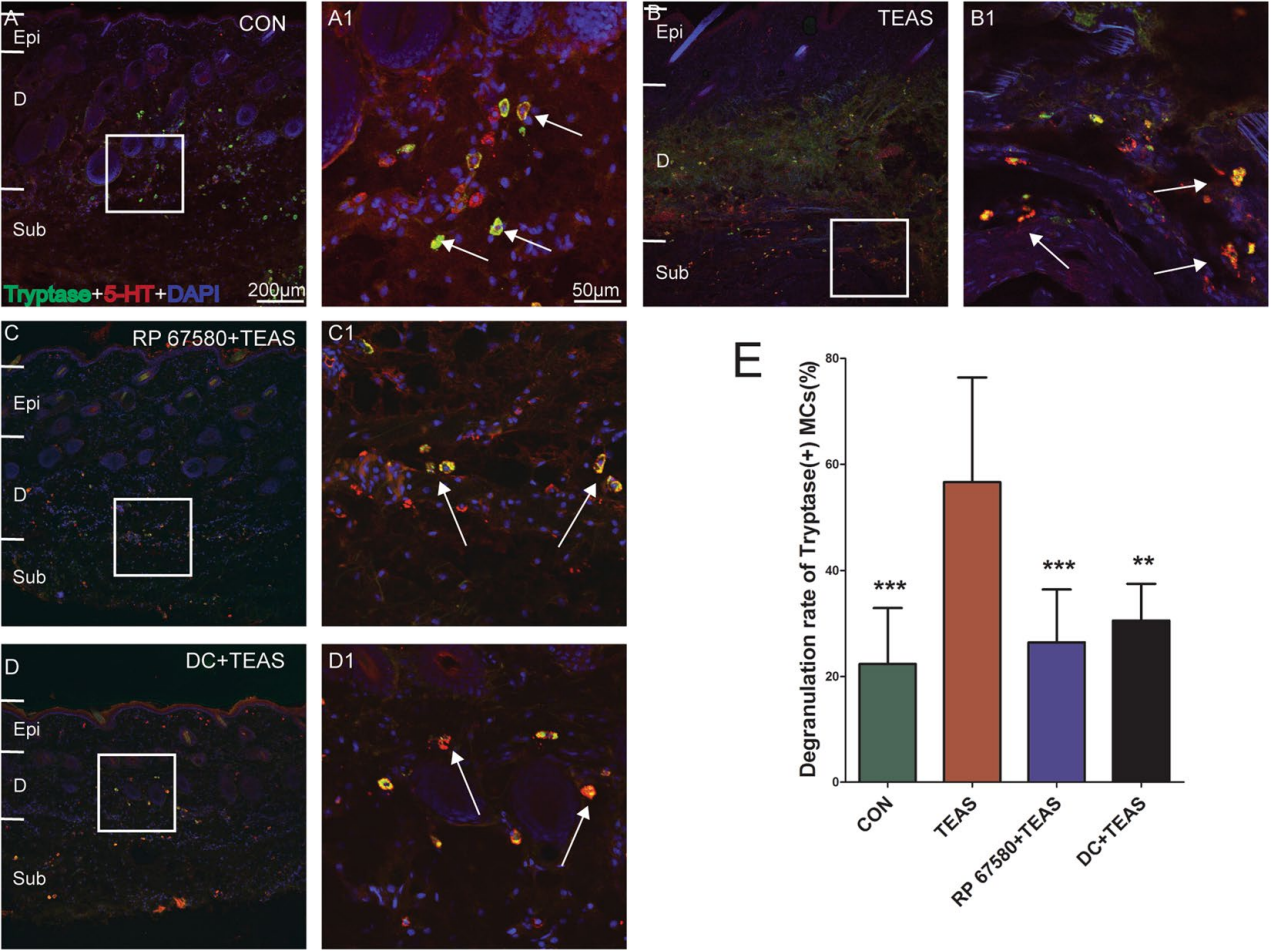
Effect of administration of NK-1R antagonist (RP 67580) and mast cell membrane stabilizer (DC) on the degranulation of the MCs induced by TEAS. In comparison with control (**A**, A1), degranulation of the MCs increased after TEAS (**B**, B1). Degnulation of the MCs induced by TEAS decreased after intraperitoneal injection of RP 67580 (**C**, C1) and DC (**D**, D1), A scale bar for (**A**–**D**) is shown in A, and that for A1–D1 is shown in A1. Epi: epidermis. D: dermis. Sub: subcutaneous tissue. (**E**) The degranulation rate on MCs induced by TEAS increasingly decreased after injection of NK-1R antagonist and disodium cromoglycate. (**P < 0.05 *vs* TEAS, ***P < 0.001 *vs* TEAS).

After DC or RP 67580 was administrated, the degranulation rates of MCs significantly decreased from 60.55% ± 15.46% (P = 0.00002) to 30.56% ± 6.922% (P = 0.0002) and 26.44% ± 9.98% (P = 0.0001) respectively in LI4 after TEAS (Fig. 9).

## Discussion

In this study, we showed that TEAS induced high expression of CGRP-IP and SP-IP nerve fibres to activate MCs by NK-1R. The activated MCs aggregated with degranulation of 5-HT near the CGRP-IP, SP-IP sensory nerve fibres and blood vessels. This response conveyed TEAS signals to certain pathways.

**Figure 10:**
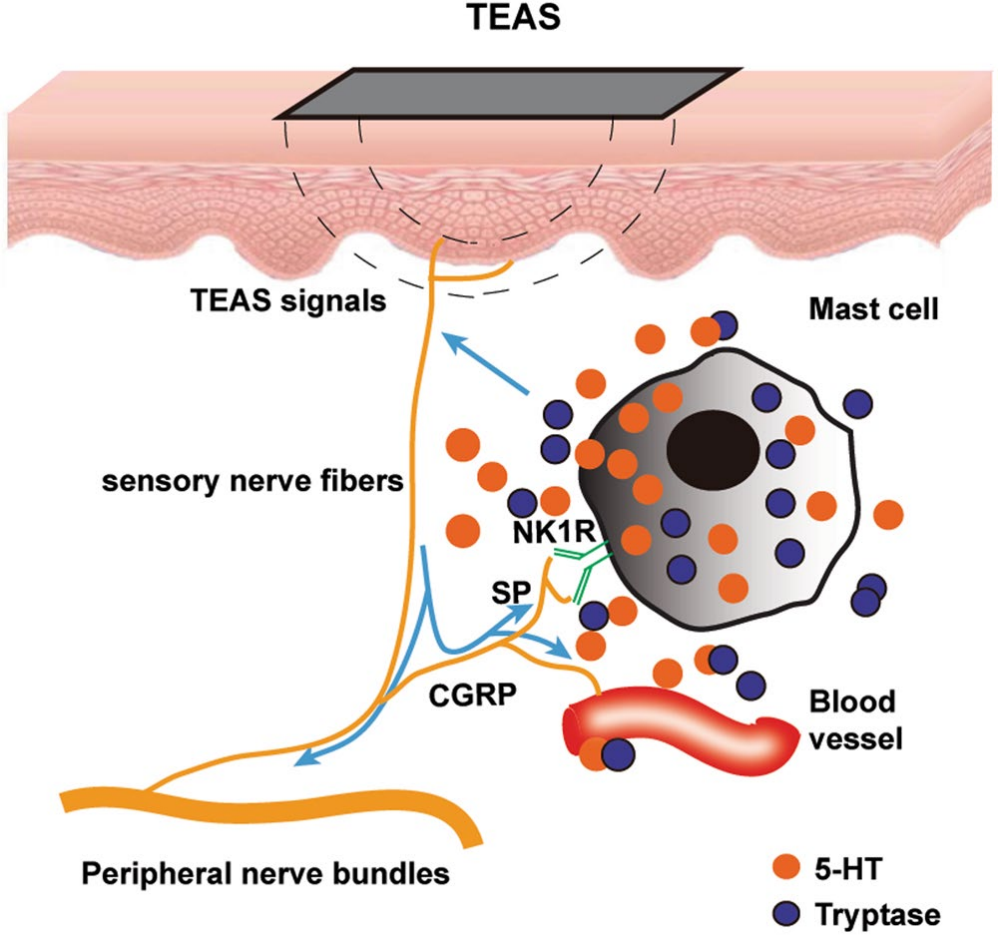
Schematic of the mechanism of TEAS on the sensory nerve-MC unit. TEAS signals are transmitted to the terminal of sensory nerve terminals with the release of SP and CGRP. SP acts on the NK-1R of the MCs via the axon reflex. The activated MCs aggregate and degranulate with 5-HT granules near the CGRP-IP or SP-IP nerve fibres and blood vessels.

TEAS is a handy therapy for patients to continue treatment at home. The mechanism of TEAS is important for its promotion. Previous studies showed that needling reactions included neuronal, biophysical and biochemical components. All types of somatic afferent nerve fibres (I-IV) were activated by MA stimulation, eliciting various effects^3,18^. MC activation at acupoints was important for the EA effect against pituitrin-induced bradycardia in rabbits^19^. The degranulation of MCs participated in the effects of acupuncture^19,20^, moxibustion^21^, and laser acupuncture^22^. Our previous study^11^ showed that MA stimulation induced high expression of nociceptive neuropeptides of SP and CGRP in subepidermal nerve fibres to activate MCs. MCs aggregated and degranulated, releasing tryptase, 5-HT and HA. These neuroactive components played a key role in conveying acupuncture signals to various pathways to deliver the effects of acupuncture. Present data provided evidence that TEAS also induced MCs aggregation and degranulation with release of 5-HT around the sensory nerve fibres and blood vessels (Fig. 10), suggesting that TEAS and MA induced the same dermal and sub-dermal changes at the same acupoint (LI4).

Neuropeptide receptors NK-1R and NK-2R were found to be largely localized on MCs, and the increased expression of NK-1R and NK-2R on MCs played a role in the MC-nerve association^23^. SP was a neurotransmitter in relation to MCs activation by NK-1R^17^ and this action can be diminished by application of NK-1R antagonist^24^. NK-1R antagonists bind to NK-1R on the MC, resulting in competitive or noncompetitive inhibition of the SP/NK-1R signalling pathway. In the present study, we observed that expression of NK-1R increased in MCs and MCs were activated by TEAS. The activation was diminished by NK-1R antagonist or mast cell membrane stabilizer. It indicated that SP receptor NK-1 on MCs played an important role in local response at LI4 of TEAS, which strongly supported the hypothesis.

TEAS is “acupuncture-like TENS” and ideally combined acupoint and bioelectricity. It should be noted that electrical stimulation can also modulate MCs secretion directly. Electrical stimulation of the cholinergic hypoglossal nerve caused a progressive degranulation and there was a close anatomical association between MCs and pre-terminal axons. The distance between the plasma membranes of the MCs and pre-terminal axons was less than 100 nm and in some points they seemed to be in contact^25^. Therefore, not only the cholinergic nerve but also sensory nerve by electrical stimulation activates the MCs, which indicates that activation of the nerve fibres is important in initiating the local response of TEAS. Recent studies proposed that a mechano-transduction pathway participated in MC-nerve cell interactions at the acupoint. MCs also transduced mechanical stimuli to acupuncture signals by activating histamine H1 and adenosine A1 receptors^26^ through TRPV2 channels. This results suggested that the MC-nerve functional unit was a key component of physiologic and pathophysiologic responses^27^, especially after TEAS. Further study will focus on TEAS activating the MC-nerve functional unit to regulate the pathophysiologic conditions.

Previous study identified that both CGRP and SP participated in MC-nerve associations^23^. In the present study, we found that TEAS activated the expression of both CGRP and SP in sensory nerves. The role of SP and its receptor on MCs were investigated in the local response to TEAS. Further study should be done to elucidate CGRP-MC crosstalk in local response of TEAS.

The procedure of MA therapy involves insertion and manipulation of the needles under skin to regulate Qi. The needles can enter either into muscles, near vascular walls or even into periostea to cause various feelings in patients^28^. Therefore, the local effects of acupuncture depend on the local tissues of acupoints as well as acupuncture manipulations, including twirling, lifting and thrusting needles. There are various approaches of acupuncture manipulations, and they are highly dependent on acupuncturist technique and subjective feelings of both doctors and patients. Generally, twisting at muscle-rich acupoints produces stronger distant effects than those produced by cutaneous receptors^29^. Obviously, TEAS has limited clinical use. It is necessary to develop a new TEAS instrument that can replace acupuncture by mimicking manual manipulation in various tissues layers to achieve significant effects. Further studies on more reactions triggered by TEAS in various tissues are encouraged in the future, and more evidence is needed to prove whether EA or MA can be replaced by TEAS.

## Conclusion

TEAS induced nerve fibres expressing neuropeptides CGRP and SP, SP activated the MC via binding to its NK-1R; activated MCs released 5-HT to convey acupuncture-like signals to certain pathways.

## Data Availability

The datasets generated and/or analysed during the current study are available from the corresponding author upon reasonable request.
